# Ancient Adversary – HERV-K (HML-2) in Cancer

**DOI:** 10.3389/fonc.2021.658489

**Published:** 2021-05-13

**Authors:** Eoin Dervan, Dibyangana D. Bhattacharyya, Jake D. McAuliffe, Faizan H. Khan, Sharon A. Glynn

**Affiliations:** ^1^ Discipline of Pathology, Lambe Institute for Translational Research, School of Medicine, National University of Ireland Galway (NUIG), Galway, Ireland; ^2^ Laboratory of Cancer ImmunoMetabolism, National Cancer Institute, National Institutes of Health, Frederick, MD, United States

**Keywords:** HERV human endogenous retroviruses, cancer, metastasis, epigenetics, biomarker, transposable element (TE)

## Abstract

Human endogenous retroviruses (HERV), ancient integrations of exogenous viruses, make up 8% of our genome. Long thought of as mere vestigial genetic elements, evidence is now accumulating to suggest a potential functional role in numerous pathologies including neurodegenerative diseases, autoimmune disorders, and multiple cancers. The youngest member of this group of transposable elements is HERV-K (HML-2). Like the majority of HERV sequences, significant post-insertional mutations have disarmed HERV-K (HML-2), preventing it from producing infectious viral particles. However, some insertions have retained limited coding capacity, and complete open reading frames for all its constituent proteins can be found throughout the genome. For this reason HERV-K (HML-2) has garnered more attention than its peers. The tight epigenetic control thought to suppress expression in healthy tissue is lost during carcinogenesis. Upregulation of HERV-K (HML-2) derived mRNA and protein has been reported in a variety of solid and liquid tumour types, and while causality has yet to be established, progressively more data are emerging to suggest this phenomenon may contribute to tumour growth and metastatic capacity. Herein we discuss its potential utility as a diagnostic tool and therapeutic target in light of the current *in vitro*, *in vivo* and clinical evidence linking HERV-K (HML-2) to tumour progression.

## Introduction

The estimated 21,000 protein-coding genes account for a mere 1-2% of the approximately 3 billion base pairs comprising the human genome. 20% of non-coding regions co-ordinate gene expression by directly governing transcription (promoter and enhancer regions, docking sites for epigenetic modifying factors) or producing non-coding regulatory RNAs such as micro RNAs (miR) and long non-coding RNAs (lncRNA). Astonishingly, 45% of our chromosomal DNA is in fact not human at all, but transposable element (TE) derived sequences that laid siege to our genome throughout evolution (1). First discovered by Barbara McClintock in maize, TEs, also known as “jumping genes” or transposons, are mobile genetic elements that can change position within their host genome (2). Infection of germ cells and subsequent fertilisation resulted in their stable incorporation and “immobilisation” within human chromosomal DNA. Following fixation in their host DNA they are transmitted vertically by Mendelian inheritance. Two classes of TEs, delineated by their method of integration and means of transposition are present in humans. Class I is composed of retrotransposons that utilise a “copy and paste” mechanism i.e. they produce self-copies *via* an RNA intermediate, thus allowing propagation within the host. Class I can be subdivided into non-long terminal repeat (LTR) retrotransposons, e.g. long and short interspersed nuclear elements (LINE and SINE), and LTR retrotransposons that include mammalian-apparent LTR-retrotransposons (MaLR) and human endogenous retroviruses (HERV). It should be noted that HERVs do not “jump” like other TEs, rather additional copies are inserted into the genome when retro-transposition of reverse transcribed RNA occurs.

Class II encompasses DNA transposons that transpose by the excision of their DNA template and subsequent integration into new genomic regions (3). Long thought of as mere vestigial genetic elements, we are now beginning to appreciate the important role TEs have played in our evolution, understand their functional role during development, and uncover their more sinister pathophysiological consequences.

## Human Endogenous Retrovirus-K (HML-2)

Endogenous retroviruses (ERV) are the result of stable integrations of exogenous retroviruses into vertebrate genomes. ERVs came to light in the late 1960’s and early 1970’s with the discovery of the avian leukosis virus (ALV), murine leukaemia virus (MLV), and mouse mammary tumour virus (MMTV) (4). The first human endogenous retrovirus (HERV) was identified in 1981 (5). These “fossil viruses” account for ~8% of our DNA (1, 6). HERVs are subclassified based on the phylogenetic distinction of their exogenous ancestors. Class I is composed of *gammaretrovirus*-like insertions, class II of *betaretrovirus*-like insertions, and class III of *spumaretrovirus*-like insertions (7). Their nomenclature is contentious and often confusing with multiple designations ascribed to individual families and their members (8). HERV integrants are most commonly named after the presumptive transfer RNA (tRNA) used for priming of viral RNA reverse transcription. The human endogenous retrovirus-K (HERV-K) clade are, therefore, named as a result of their use of a lysine (K) tRNA (9). Ono et al., identified the HERV-K family and reported the first complete HERV-K sequence in 1986 (10). Members of the *betaretrovirus*-like elements, the HERV-K supergroup is now known to consist of eleven subgroups, HML1-11. The HML (human mouse mammary tumour virus-like) designation owes to their close relationship to the MMTV which is responsible for the vertical transmission of murine breast cancer (10–12). The youngest (and therefore, least defective) HERV-K members are that of the HML-2 subgroup, which may have integrated as recently as 250,000 years ago (13). HML-2 can be further segregated into three clusters based on their LTR sequences; LTR5A, LTR5B, and LTR5Hs. LTR5Hs elements integrated most recently and include 28 members absent in non-human primates (14). Like all TEs, HML-2 proviral insertions have been truncated due to extensive mutations (missense, nonsense, frameshifts), deletions and recombination events post-endogenization. Consequently, no fully intact, replication competent, and infectious provirus remains. Nonetheless, concomitant re-activation of multiple HML-2 insertions has the potential to produce infectious viral particles. *In silico* reconstitution of a likely HML-2 ancestral consensus sequence rendered an infectious virus capable of *de novo* chromosomal insertions (15). HML-2 viral-like particles (VLP) have been observed *in vitro* (16). In contrast to other HERVs, The HML-2 subgroup has retained copies of open reading frames (ORF) for all its constituent proteins, and, due to a marked “reawakening” in a broad variety of solid and liquid tumour types, has been of particular focus in cancer biology. However, whether HML-2 plays a causal role in carcinogenesis or is simply an ancillary event remains unknown, and is a matter of controversy in the field.

**Figure 1:**
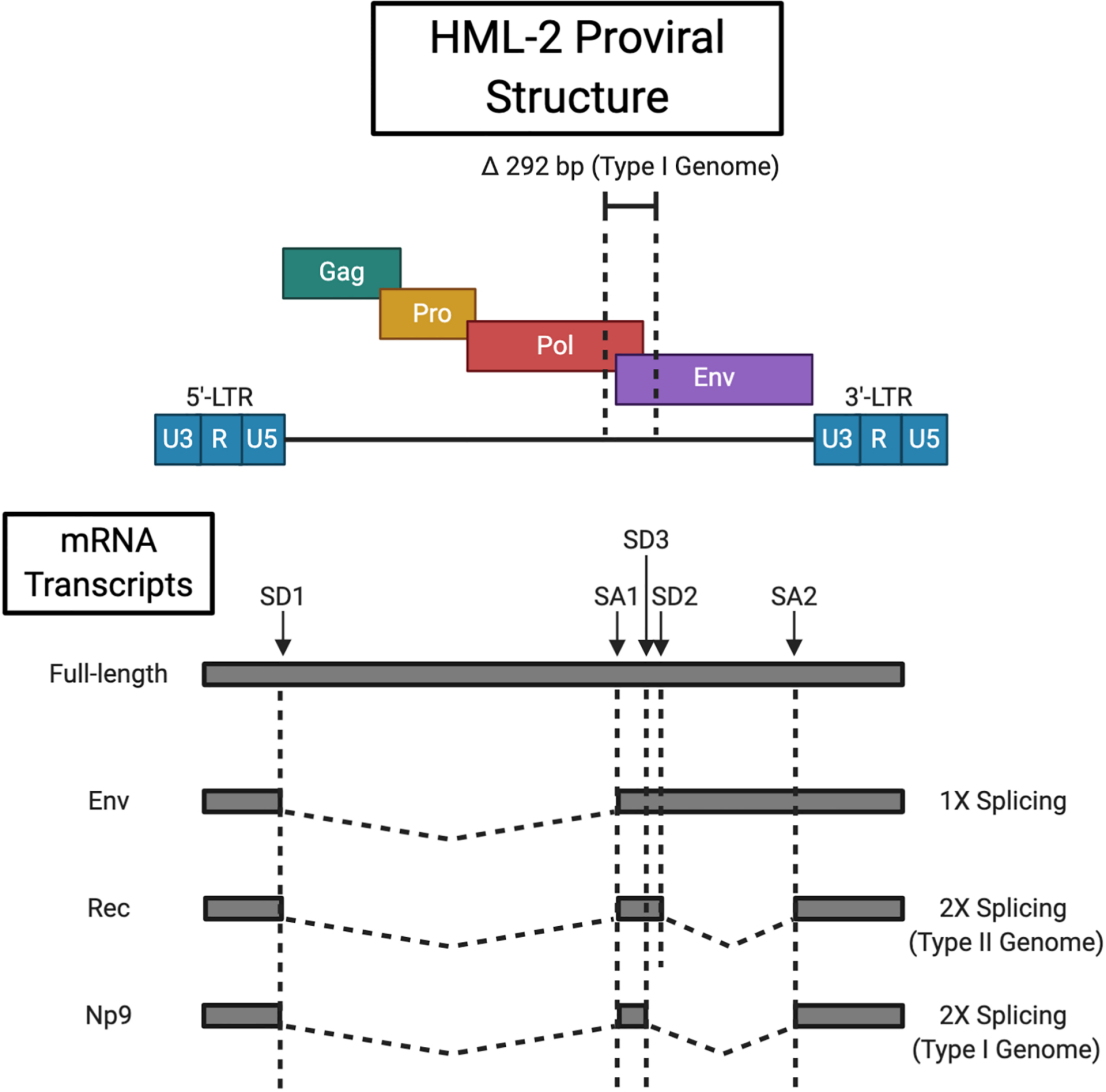
HML-2 Proviral Structure. HML-2 proviral integrations have either a type I or type II genome delineated by the respective presence (type I) or absence (type II) of a 292 bp deletion at the *pol/env* boundary. The full length transcript codes for the Gag precursor and Gag-Pro/Gag-Pro-Pol fusion proteins that are processed by the viral protease and cellular proteasome respectively. Singly sliced HML-2 mRNA encodes the Env protein, while a secondary slicing event of *env* mRNA gives rise to the Rec (type II) or Np9 (type I) accessory proteins. Splice donor (SD) and splice acceptor (SA) sites are indicated (type II loci combine SD2 and SA2 for *rec* splicing while type I loci contain the alternative SD3 site for *np9* transcript generation). Created with BioRender.com.

HERVs resemble their exogenous relatives (e.g. HIV) in structure. The core viral genes, *gag*, *pro*, *pol* and *env* are flanked by two LTR sequences (see **Figure 1**). The 5’-LTR functions as a promoter region to drive proviral gene expression, however, read-through may occur from adjacent promoters (17). While shown to lack canonical promoter elements such as a TATA box or initiator (Inr) element, *in silico* analysis has predicted multiple consensus binding sites for a broad range of transcription factors (TF) (18, 19). Transcription initiates at the transcription start site (TSS) located between the 5’ R and U5 regions, and is terminated by the 3’-LTR poly-adenylation signal (20). Expression is at least partially regulated by the Sp1 and Sp3 TFs that recognise specific GC box motifs and facilitate transcription pre-initiation complex assembly (19). Although inactivating mutations are favoured by selective pressures, activating polymorphisms in TF binding sites have been identified, which may in part account for inter-individual variability in HML-2 expression (21). 91 HML-2 sequences with varied coding capacity are littered throughout the human genome (22). HML-2 solo-LTRs, generated following homologous recombination between the 5’ and 3’ LTRs, are present at about 10-times the frequency of known insertions with internal coding regions (22). HML-2 is initially transcribed as a full-length transcript that encodes the Gag (group-specific antigen), protease (Pro) and polymerase (Pol) proteins. -1 base pair (bp) ribosomal frameshifting at two slippery sites within the full-length mRNA allows for the production of Gag, Gag-Pro, and Gag-Pro-Pol fusion proteins (23). The vast majority (~ 95%) of translation events terminate at the Gag stop codon (24). Subsequent proteasomal processing liberates the Pro and Pol precursors. The functional 18 kDa Pro enzyme, akin to retroviral aspartic proteinases, is self-excised by autocatalytic cleavage (22, 23). The antecedent 79 kDa Gag polyprotein is cleaved by the active Pro into the mature structural components, i.e. the matrix (MA, p15), capsid (CA, p27), the nucleocapsid (NC, p10), three smaller peptides, and a putative phosphoprotein (pp15) (25). Pol reverse transcribes the full-length proviral RNA as well as providing integrase (IN) activity for host-genome insertion (26). A single splicing event generates the *env* transcript that gives rise to the envelope (Env) glycoprotein. Viral Env proteins encapsulate the virus and facilitate cellular entry by recognising a virus-specific cognate receptor (the HML-2 Env receptor remains unidentified) (27). The pre-protein consists of a 13 kDa N-terminal signal peptide (SP), 55 kDa surface unit (SU), and 39 kDa transmembrane (TM) unit. The SP guides the immature Env to the endoplasmic reticulum (ER) for N-linked glycosylation and is then removed by signal peptidases (28). Env precursor homotrimers are cleaved by furin-like proteases (at a consensus R-X-K/R-R motif) in the late Golgi apparatus to yield the functional SU and TM subunits that associate non-covalently (29, 30). A secondary splicing event of the *env* mRNA generates the *rec* transcript that codes for the nucleo-cytoplasmic shuttle protein Rec (formally designated cORF) (31, 32). Rec functionally (but not structurally) resembles the HIV Rev and HTLV-1 Rex proteins. It binds Rec responsive elements (termed RcRE for “regulatory cORF-responsive element”) within the 3’ untranslated region (3’-UTR) of nascent HML-2 mRNAs, and expedites their nuclear export *via* the chromosomal region maintenance 1 (CRM1) pathway (33, 34). This serves to direct a balanced translation of full-length and spliced transcript species. The active Rec complex consists of three Rec tetramers that recognise an intricate stem-loop RNA secondary structure as opposed to a discrete sequence (35, 36). However, Rec is not limited to binding HML-2 transcripts having been demonstrated to engage with other cellular RNAs and upregulate their ribosomal occupancy (37). Two types of HML-2 sequences distinguished by the presence/absence of a 292 base pair (bp) deletion at the *pol-env* boundary are resident in the human genome. Type I elements harbour this deletion and consequently express an alternative accessory protein to the type II Rec, nuclear protein of 9 kDa (Np9) (35). Np9 viral function is thus far undefined. Additionally, the type I deletion causes an in-frame fusion of the *pol* and *env* open reading frame, the consequences of which on Pol function are currently unknown (38).

As noted above, a subset of HML-2 insertions are human specific (14). One could, therefore, speculate that HML-2 has contributed to speciation and human genome divergence during the human-chimpanzee split. Further to this some loci are polymorphic within the global population. Several groups have reported unfixed elements present in only a subset of individuals (13, 39, 40). Larger cohorts are required to elucidate any association between polymorphic insertions and disease susceptibility. Such an undertaking would necessitate large scale whole genome sequencing (WGS).

## Transcriptional, Post-Transcriptional and Epigenetic Regulation of HML-2

The 5’-LTR of HML-2 insertions serves as a promoter region to drive proviral gene expression. *In silico* analysis of the 5’-LTR has predicted the presence of consensus binding sites for a broad array of transcription factors (41), while it has been described as a “landing strip for inflammatory transcription factors” (18). Functional interferon-stimulated response elements (ISREs) have been demonstrated to be present as evidenced by increased HML-2 mRNA and protein expression following TNF-α and LIGHT cytokine treatment of astrocytes and neurons. This effect was mediated by the canonical p65/p50 nuclear factor κB (NF-κB) complex and interferon response factor 1 (IRF1). Ectopic expression of both transcription factors led to the upregulation of HML-2 transcription, and subsequent chromatin immunoprecipitation (ChIP) coupled qRT-PCR demonstrated a direct binding to ISREs within the 5’-LTR (41). With inflammation being a key driver of tumorigenesis and cancer progression, the rich cytokine milieu of the TME may play a major role in HML-2 upregulation. Steroid hormones and their receptors are also known to contribute extensively to HML-2 regulation and expression. Co-treatment of hormone receptor positive breast cancer cell lines with β-estradiol and progesterone stimulated HML-2 transcription (42). Treatment of prostate cancer cell lines with dihydrotestosterone (DHT) or the synthetic analogue R1881 has been shown to result in an increase in HML-2 transcripts (43, 44). Both Goering et al. and Hanke et al. showed increased luciferase activity of reporter constructs under the control of the HML-2 5’-LTR following androgen receptor (AR) stimulation (44, 45). Further to this, Rec was demonstrated to interact with and inhibit the human small glutamine-rich tetratricopeptide repeat protein (hSGT), a co-chaperone and negative regulator of AR activity. This led to AR de-repression and the formation of a positive feed forward loop driving AR activity and HML-2 self-propagation (45). Rec also directly binds and abrogates the AR repressor activity of the testicular zinc finger protein (TZFP) *via* its N- and C-terminal domains (46). Based on these studies it is interesting to speculate that HML-2 Rec may function as an oncoprotein in prostate and testicular cancers by impeding AR repressor proteins, and its targeting could prove additive/synergistic with anti-androgen therapy.

At the post-transcriptional level, HML-2 translation may be regulated by RNA binding proteins (RBP). Fouroushani et al. examined HERV and RBP transcript expression in RNA-sequencing (RNA-seq) datasets from pre-implantation embryos and induced pluripotent stem cells (iPSC). RNA-binding motif protein 4 (RBM4) was identified to inversely correlate with HERV expression and shown to bind HML-2 RNA at CGG consensus elements. RBM4 knockout led to HML-2 transcript upregulation and increased Env protein expression in the chronic myelogenous leukaemia (CML) HAP1 cell line (47). RMB4 has been suggested as a tumour suppressor and its reduced expression is associated with poor overall survival in lung, breast, ovarian and gastric cancers (48, 49). On the other hand, the RBP Staufen-1 was shown to associate with, and act as a Rec co-factor to promote HML-2 translation and viral particle assembly. Interaction of its RNA binding domain 4 with Rec augmented the nuclear export of unspliced HML-2 transcripts with functional RcREs (50). TAR (trans-activation-responsive) DNA binding protein 43 (TDP-43), which is known to be dysregulated in cancer, was shown to bind the HML-2 5’-LTR and upregulate expression *via* the recruitment of RNA polymerase II (RNA pol II) in neurons (51).

HML-2 is largely silenced in healthy somatic tissue, however, expression is detectable (52, 53). While hypomethylation of the 5’-LTR CpG dinucleotide island has been purported as the predominant mechanism underlying HML-2 resurrection during carcinogenesis, it is not the sole determinant of transcriptional activity (54, 55). Treatment of peripheral blood mononuclear cells (PBMCs) from healthy donors with the DNA methyltransferase (DNMT) inhibitor 5-azacytidine (5-aza) did not modulate HML-2 expression (56). Kreimer et al., observed the HERV-K 22q11.23 locus to be hypomethylated in bladder cancer without an accompanying activation of transcription (57). Other epigenetic changes during carcinogenesis and the overall chromatin landscape at HML-2 loci have not been comprehensively described. However, a significant amount of information has amassed concerning HML-2 epigenetic regulation during embryogenesis which may have implications in cancer owing to the frequent hijacking of developmental transcriptional programmes by malignant tumour cells. Turelli et al., used ChIP and deep sequencing (ChIP-seq) to demonstrate a significant proportion of class II HERVs, including some HERV-K family members, were bound by TRIM28 (also known as KAP1) in embryonic stem cells (ESC). This was accompanied by the recruitment of the histone-lysine N-methyltransferase SETDB1, deposition of the repressive H3K9me3 histone mark, and consequent silencing of transcription (58). However, this mechanism may not ubiquitously regulate HERV-K members as no association was observed between KAP1 and HML-2 expression in ovarian cancer tissues (59). In a follow up paper the same group showed enrichment of the active H3K27ac chromatin mark accompanied by KLF4 and OCT4 binding at LTR5Hs elements. Interestingly, a majority of the marked regions did not correspond to a transcript, suggestive of a broader function as enhancer regions regulating the naïve ESC transcriptional network. Their findings corroborate that of Fuentes et al., who used a proprietary CRISPRi/a system to target and inhibit/activate HML-2 LTR5Hs activity. They showed that HML-2 elements are intrinsically involved in gene regulation during embryogenesis, operating as enhancer regions for neighbouring and even distal genes (>200 kb upstream or downstream) (60). The idea that HML-2 LTRs may serve as *cis*-regulatory elements by providing alternative, or even tissue specific enhancers, for protein coding genes has been directly demonstrated for *PRODH* which encodes a mitochondrial proline dehydrogenase (PRODH) enzyme. Hypomethylation of the adjacent HML-2 LTR and binding of the SOX2 transcription factor increased *PRODH* expression (61). Further to this, HML-2 and PRODH expression was shown to co-correlate with the differentiation stage of germ cell tumour (GCT) cells, being most highly expressed in undifferentiated embryonal carcinoma (EC) cells. Expression was partly rescued in differentiated GCT cells by inhibition of DNA methylation and histone deacetylation with 5-aza and trichostatin A respectively (62). These data describing a role for HML-2 loci as *cis*-regulatory elements raise the interesting possibility of their onco-exaptation for the regulation of tumour-specific gene expression.

Krüppel-associated box (KRAB) zinc finger proteins (ZFP) (KRAB-ZFPs) are represented by 423 independent loci. Alternate splicing gives rise to 742 structurally distinct proteins that appear to have co-evolved with and target TEs for epigenetic silencing. KRAB-ZFPs appear engaged in an evolutionary “arms race” with these germline predators. The KRAB-ZFP family members ZNF417 and ZNF587 have been identified as negative regulators HML-2 in ESCs (25).

The apolipoprotein B mRNA editing enzyme, catalytic polypeptide-like (APOBEC3) family are viral restriction factors that target the negative-strand retroviral DNA intermediate. Deamination of cytosine residues to uracil results in guanine-to-adenine (G→A) substitutions within the positive strand that undergoes host genome integration. HML-2 members have been shown to bear “hypermutated” footprints consistent with APOBEC3G activity (63).

HML-2 translation may be limited by its use of rare leucine (CUA and UUA) and valine (GUA) codons for which the corresponding tRNAs are less abundant in the human tRNA pool. This may limit protein translation from mRNA. Codon optimisation of HML-2 *env* enhanced translation efficiency relative to the corresponding wild-type sequences *in vitro* (64).

## HML-2 Expression in Tumour Tissues and Correlation With Clinical Characteristics

### Breast

HML-2 is expressed in numerous tumour types as summarised in **Table 1**. As previously mentioned, HML-2 expression can be regulated by progesterone and estrogen in in hormone receptor positive breast cancer cell lines (42). There have been multiple reports of HML-2 transcript and protein overexpression in tumour specimens from patients with breast cancer. HML-2 Env was overexpressed in malignant versus benign healthy tissue and associated with disease stage and increased risk of lymph node metastasis. High Env expressing patients exhibited decreased overall survival compared to patients with moderate/low expressing tumours (65). In a separate study two thirds of primary breast cancer tissues were shown to be positive for HML-2 by immunohistochemistry (IHC). HML-2 positive tumours were again associated with an increased incidence of lymph node metastases (80). Breast cancer is composed of a number of defined molecular subtypes delineated by the expression/absence of the estrogen receptor (ER), progesterone receptor (PR) and human epidermal growth factor receptor 2 (HER2). The most aggressive is the triple negative breast cancer (TNBC) subtype which lacks expression of all 3, and hence has no effective targeted therapy. Analysis of TCGA RNA-seq data from patients with invasive ductal carcinoma (IDC) revealed an upregulation of HML-2 in basal-like breast cancers (which largely comprises those of the TNBC molecular subtype) compared to HER2 overexpressing and luminal tumours. HML-2 expression positively correlated with cyclin-dependent kinase 6 (CDK6), E2F Transcription Factor 5 (E2F5) and phosphorylated (i.e. inactive) retinoblastoma protein (pRb) expression, which may indicate a role in cell cycle regulation (66). While mRNA and protein levels don’t always necessarily match (true for many genes), there does tend to be a trend in that direction.

**Table 1:** Summary of HML-2 alterations detected in patient specimens and their associated clinical parameters.

Cancer Type	Observed Change in HML-2	Clinical Association	Reference
**Breast**	↑ Env transcript and protein expression	↑ Disease stage, lymph node metastasis, ↓ overall survival	(65)
	↑ transcript expression	↑ in basal-like vs HER+/luminal tumours	(66)
	↑ serum HML-2 mRNA	Early marker of metastatic risk	(67, 68)
**Prostate**	↑ HERV-K_22q11.23 5’LTR-*gag* and *env*		(44)
	↑ HERV-K_22q11.23 Gag		(43)
	↑ *gag* mRNA in PBMCs	↑ odds of prostate cancer diagnosis	(69)
	↑ Gag autoantibodies	Correlated with clinical stage, Gleason score, eventual metastasis, worse overall survival and faster recurrence	(43)
**Melanoma**	↑ Env protein		
	HML-2 5’-LTR hypomethylation	Lymph node positivity, ↑ tumour stage, ↑ risk of recurrence, ↓ disease-free survival	(70)
	↑ Env and Gag autoantibodies	Acrolentiginous/mucosal/uveal subtypes, associated with disease stage and ↓ survival probability	(71)
**Lung**	↑ *env* mRNA in blood	Distinguished adenocarcinoma from squamous cell and small cell lung cancers	(72)
**Hepatocellular**	↑ transcript expression	Associated with liver cirrhosis, tumour differentiation, TNM stage and worse overall survival	(73)
**Colorectal**	HML-2 5’-LTR hypomethylation		(74)
	↑ Env protein		(75)
**Soft Tissue Sarcoma**	↑ transcript expression	Worse relapse-free survival	(76)
**Leukaemia**	↑ Np9 mRNA and protein		(77, 78)
**Lymphoma**	↑ transcript expression	Transcript expression decreased with treatment	(79)

### Prostate and Genitourinary

Similar to breast cancer, HML-2 has been found to be androgen inducible in prostate cancer cell lines. Exploring HML-2 mRNA levels in prostate cancer specimens revealed significantly elevated expression of HERV-K_22q11.23 5’LTR-*gag* and *env* in 45 prostate cancer tumour samples versus 11 benign prostate tissues. A more discriminate pattern was observed in the case of the accessory protein *Np9*, with only a subset of carcinomas exhibiting expression of its transcript. Interestingly HERV-K17 expression was diminished in cancerous versus benign samples, while HERV-K_11q23.3 was undetectable as seen in prostate cancer cell lines (44). In contrast, Reis et al. detected HERV-K_22q11.23 *gag* expression in 11 of 11 prostate cancer tissues. Tissue microarray (TMA) analysis showed that 159/188 prostate cancer samples exhibited HERV-K_22q11.23 Gag immunoreactivity while only 9/22 normal samples were weakly positive. All 12 metastatic samples examined tested positive (43). Aberrant expression of HML-2 Env protein has been detected in prostate cancer tissues but not in cases of benign prostatic hyperplasia (BPH) (69). Theoretically HML-2 could contribute to tumorigenesis by insertional mutagenesis and chromosomal rearrangements. While the former is unlikely as it would require an intact provirus there has been at least one report of a HML-2 LTR translocation event. Fusion of the *HERV-K_22q11.23* promoter to the oncogenic E26 transformation specific (ETS) family member ETV1 was shown to confer androgen responsiveness and drive its overexpression in a subset of prostate cancers (81). HML-2 upregulation is a common event in germ cell tumours (31). The putative oncogene *rec* may be implicated in seminomagenesis. Inducible Rec overexpression disrupted germ cell development leading to carcinoma *in situ*-like features in mouse testes (82). Ectopic Rec expression in Rat-1 fibroblasts conferred tumour-forming capacity *in vivo via* inhibition of the promyelocytic leukaemia zinc finger protein (PLZF), further underlying its potential involvement in cellular transformation (83).

### Melanoma

Multiple studies have detected the presence of HML-2 in melanoma (84). Studies suggest a role for UV light in HML-2 activation in normal human epidermal melanocytes (85), while there are reports of UV light either promoting (86) or inhibiting HML-2 in melanoma cell lines (85). In clinical specimens HML-2 Env protein was detected in melanoma but not in benign tissue from melanoma patients (87). Cardelli et al. reported HML-2 5’-LTR hypomethylation in melanoma tissues when compared to benign nevi. A decreased methylation profile correlated with other prognostic features including lymph node positivity and higher tumour stage. Lower methylation was associated with an increased risk of recurrence (ROR) and shown to be an independent predictor of decreased disease-free survival (DFS) (70). There is also evidence for a humoral response against HML-2 proteins in melanoma. Hahn et al. detected anti-HERV-K Gag and Env antibodies in a subset of melanoma patients but not in healthy controls, particularly those with non-UV associated acrolentiginous/mucosal/uveal melanoma patients. Patients with anti-HML2 antibodies had a significantly increased risk of poor outcome independent of stage at diagnosis (71).

### Other Solid Tumours

In a study of 60 lung cancer patients and 20 healthy controls, Zare et al., detected a 10-fold increase in HERV-K *env* mRNA in patient blood samples. While HERV-K expression levels could distinguish adenocarcinoma from squamous cell carcinoma and small cell lung cancer patients, there was no association with disease stage (72). qRT-PCR analysis of HML-2 revealed increased transcript expression in hepatocellular carcinoma (HCC) tissues relative to the adjacent healthy tissue. Expression was associated with liver cirrhosis, tumour differentiation and TNM stage, while highly expressing patients had worse overall survival (Hazard ratio =3.07) (73). The role of HML-2 in colorectal cancer is not completely understood. While Dolci et al. found HML-2 LTRs to be hypomethylated in colorectal cancer (CRC) tumours compared to adjacent healthy tissue, the *env* gene was not observed to be upregulated (74). A follow-up study failed to recapitulate the finding of LTR hypomethylation in tumour samples relative to matched healthy tissue, however, Env protein expression was observed exclusively in malignant samples. Interestingly, and in contrast to most data pertaining to HML-2 expression in cancer, Pol protein expression was significantly higher in healthy tissues versus the matched tumour samples (75). Elevated HML-2 transcript expression corelated with shorter relapse-free survival in soft tissue sarcoma patients but showed no association with overall survival (76).

### Haematological Malignancies

HML-2 mRNA has been observed to be upregulated in leukaemia versus healthy PBMCs (56). Fischer et al. found increased *Np9* but not *gag* transcript expression in chronic lymphocytic leukaemia (CLL) which may be indicative of the selective reactivation of type I loci or simply the derepression of loci devoid of an intact *gag* ORF (77). Np9 protein expression was shown to be restricted to a subset of leukaemia patient samples (28/50 – mixed leukaemia patients) and only detected in one of 22 healthy CD34+ haematopoietic stem cell samples tested in a separate study (78). Some evidence also exists for HML-2 in Hodgkin lymphoma, with expression seen in Hodgkin lymphoma cell lines (88), and in patients with HIV associated Hodgkin lymphoma (79). Elevated HERV-K was also seen in both HIV and non-HIV associated non-Hodgkin lymphoma. Transcript levels were found to dramatically decrease in patients who went into remission following treatment (79).

## Potential as a Non-Invasive Biomarker for Disease Diagnosis, Stratification and Monitoring

Early detection and intervention significantly improves patient outcomes. Liquid biopsies have become promising tools for cancer screening, diagnosis and the monitoring of disease progression and response to therapy. Besides being minimally invasive, the detection of molecular markers of disease in blood or urine could provide a rapid and cost-effective means of patient assessment. Robust indicators of cellular transformation and tumour aggressiveness are required to help realise the potential this approach has to aid clinical decision making (89).

### HML-2 mRNA

The detection of HML-2 mRNA in blood could serve as a highly specific marker of disease in multiple cancers. HML-2 *env* was found to be significantly higher in the blood of patients with primary breast cancer while chemotherapy reduced expression levels (67). Wang-Johanning et al., showed serum HML-2 mRNA to be upregulated in women with early stage ductal carcinoma *in situ* (DCIS). Furthermore, it was found that HML-2 burden may be an early marker of metastatic risk (68). Prostate specific antigen (PSA) is the gold standard biomarker used in prostate cancer screening. While PSA is overexpressed in prostate cancer, its utility is limited given that (i) it cannot discriminate between indolent and aggressive disease (ii) specificity decreases with age, and (iii) overexpression is not restricted to malignant tumours giving rise to false positives. Wallace et al., investigated the potential use of HML-2 detection in PBMCs as a surrogate/combined test. They observed that HML-2 *gag* mRNA was significantly upregulated in prostate cancer cases versus controls, and that HML-2 burden correlated with increased odds of diagnosis. Additionally, and in contrast to PSA, the predictive ability of HML-2 *gag* was most robust in older men (69).

### Autoantibodies

Autoantibodies may be generated during the early stages of tumorigenesis before clinical symptoms manifest. Their broad applicability and current state of the art is reviewed in (90). A lack of HML-2 immune tolerance is evidenced by the detection of autoantibodies at significantly higher titres and frequencies in several cancer types compared to healthy individuals. The elicitation of a humoral immune response is a further indication of the suppression of HML-2 expression during ontogenesis and in mature healthy somatic tissues. Moreover, this supports their use as a disease-specific biomarker and continued research into HML-2 targeting immunotherapeutics. Ishida et al., first identified HML-2 Gag immunogenicity in prostate cancer. An antibody response was detected in a subset of prostate cancers (as well as in subsets of bladder, liver, lung and ovarian cancers and malignant melanoma) but absent in healthy donors (91). Serum antibodies against HML-2 Gag were separately detected at a higher frequency in prostate cancer patients when compared to healthy donors, and also shown to be more common in advanced disease (stage III-IV) versus early stage tumours (stage I-II). Gag seroreactivity correlated with clinical staging, Gleason score and eventual metastasis. The presence of anti-Gag antibodies was predictive of worse overall survival and faster recurrence (43). Rastogi et al. included anti-Gag antibodies in a panel alongside ERG and AMACR autoantibodies. This combination conferred enhanced assay sensitivity in differentiating patients from healthy controls when compared to each target alone (92). Another independent and more recent study also identified HML-2 Gag as being immunogenic in prostate cancer and found anti-Gag antibodies to be enriched in prostate cancer patient sera versus healthy donors (93). No association was found between antibody prevalence and overall survival, however, in contrast to the study by Reis et al., the cohort analysed was composed exclusively of advanced metastatic castration resistant prostate cancer (mCRPC) patients. mCRPC is non-responsive to anti-androgen therapy and incurable, therefore, targeting HML-2 may represent an enticing new immunotherapeutic approach in this setting. Significantly higher anti-Env antibody titres were detected in early stage breast cancer patient sera than in healthy donors. The area under the curve (AUC) values obtained for the assay were comparable to that of a mammogram, the standard population screening method (68).

## Functional Role of HML-2 in Tumour Progression

### Tumour Cell Proliferation and Migration

As discussed in Section 4, HML-2 expression is a potentially highly specific marker of cellular transformation and is associated with disease progression in an array of cancer types. Rather than their increased expression simply being an epiphenomenon and bystander effect of carcinogenesis, a functional role for HML-2 proteins is beginning to be appreciated. However, the molecular mechanisms underlying their impact remain largely unexplored. HML-2 Env has been shown to promote the proliferation of breast cancer and pancreatic cancer cells *in vitro* and in mouse xenograft models (94, 95), while Np9 was observed to support the growth of leukaemia and teratocarcinoma cells (78, 96). Overexpression of Np9 in leukaemia cell lines led to the activation of ERK, AKT and Notch1 signalling as well as β-catenin upregulation. The observation that HML-2 is often upregulated in aggressive versus indolent disease and in metastatic versus primary tumour samples is suggestive of a role in metastasis. Knockdown of HML-2 Env in a panel of breast cancer cells inhibited migration and invasion and was associated with the perturbation of the *Ras/Raf/MEK/ERK* signalling axis (94). Knockdown/overexpression Np9 decreased/increased NCCIT teratocarcinoma cell migration in wound healing assays (96).

### Immune Modulation/Evasion

HERV proteins, including HML-2 Env and Gag, are recognised as non-self epitopes. However, similar to its exogenous relatives, HML-2 Env may possess immunomodulatory capacity (97). The transmembrane (TM) subunit of the HML-2 Env protein harbours a putative immunosuppressive (ISU) domain. Treatment of PBMCs with teratocarcinoma cell line-derived HML-2 viral particles or a recombinant TM/ISU peptide inhibited proliferation. Cytokine secretion was altered with the immunosuppressive interleukin-10 (IL-10) being particularly increased (98). This suggests a direct interaction of Env with tumour infiltrating immune cells that may contribute to the immunosuppressive tumour microenvironment (TME) and help facilitate immune system subversion. Whether this occurs *via* a cognate receptor or modified cell signalling following endocytosis requires elucidation. This function may have been adapted to provide immune protection during foetal development with expression observed in the villous and extravillous cytotrophoblast cells of the placenta (99). While the retention of HERV sequences in the human genome suggests the development of some level of tolerance and/or domestication for normal physiological function, their silencing is indicative of persistent deleterious effects. HML-2 proteins remain antigenic and have been linked to autoimmune and neurodegenerative disorders [reviewed elsewhere (100, 101)]. This begs the question if HML-2 is involved in promoting disease-associated chronic inflammation? HERV-derived dsRNA has been reported to be sensed by Toll-like receptor 3 (TLR3) and mitochondrial antiviral signalling protein (MAVS) to stimulate a robust interferon response (102, 103). HML-2 RNA was specifically shown to bind human Toll-like receptor 8 (TLR8) and murine Tlr7 leading to canonical NF-κB (p50/p65) pathway activation (104).

### Cancer Stem Cells

Cancer stem cells (CSCs), also known as tumour initiating cells (TIAs), are a putative rare and aggressive tumour subpopulation (estimated to constitute <0.05% of solid tumours but higher in frequency in undifferentiated and liquid tumours). They possess extensive self-renewal capacity and chemoresistance, and are thought to be largely responsible for driving repopulation and tumour growth during disease relapse (105). HML-2 expression has been identified as a specific marker of ESCs and iPSCs, while expression is rapidly silenced upon differentiation (106). Owing to this, HML-2 has been suggested and investigated as a CSC marker and may represent an actionable target to aid in CSC eradication. When cultured in stem-cell medium melanoma cells exhibited enhanced stemness features (including increased CD133 positivity, Oct4 expression, colony formation, migration and invasion capacity) and an accompanied increase in HML-2 transcription. Knockdown of HML-2 prevented the expansion of the putative CD133+ CSC population (107). Np9 protein levels correlated with the number of stem/progenitor cells in leukaemia patient samples but was undetectable in CD34+ hematopoietic stem cells from healthy donors (78).

## HML-2 as a Therapeutic Target

HERV-targeted agents could provide a new class of therapies in the anti-cancer armamentarium, with broader applicability in autoimmune and neurodegenerative disorders. Considering HML-2 is upregulated in a variety of tumour types and correlates with disease progression and poor outcomes, HML-2 proteins are appealing novel targets in aggressive metastatic disease. Env-targeting antibodies induced apoptosis and inhibited breast cancer cell growth *in vitro* and tumour growth in an MDA MB 231 cell xenograft model of TNBC (80). While still in their infancy, autologous chimeric antigen receptor T (CAR-T) cell therapies have been approved for, and proven highly efficacious in, some haematological malignancies (108). Engineered cell-based technologies are advancing rapidly, therefore, new, disease-specific targets are in constant pursuit. HML-2 (and other TE-derived proteins) could provide a reservoir of tumour-associated antigens (TAA). Anti-Env CAR-T cells were shown to specifically target breast cancer cells *in vitro* with their impact being attenuated by HML-2 knockdown. Xenograft growth and metastasis were suppressed concomitant with the downregulation of p53 and upregulation of mouse double minute 2 homolog (MDM2) and phospho-ERK (109). Notably, Np9 inhibits MDM2 E3 ubiquitin ligase activity towards p53 *via* a direct binding interaction of its C-terminal domain (110). Against a backdrop of gain-of-function (GOF) mutations in the *TP53* gene, Np9-mediated p53 stabilisation could help drive malignant progression (111). Anti-Env CAR-T cells were also shown to be effective in a preclinical model of melanoma. Co-inoculation of anti-Env engineered T cells upon xenograft transplantation prevented tumour growth and metastasis *in vivo* (87). The natural anti-tumour immune response/immune signalling could potentially be stimulated by HML-2 (and other TE) upregulation following epigenetic therapy. DMNT inhibition is a promising anti-cancer approach already established in the treatment of myelodysplastic syndrome (MDS) and acute myeloid leukaemia (AML) (112). As discussed in section 3, DNA methylation, at least in part, regulates HML-2 expression. Treatment with 5-aza stimulated a type I interferon (IFN) response and apoptosis in ovarian cancer *via* TLR3 and MAVS sensing of HERV-derived double stranded RNA. DNMT inhibition also sensitised a murine model of melanoma to ant-CTLA4 immune checkpoint inhibition (102). The action of the more potent DNMTi 5-aza-2’-deoxycytidine (decitabine) in CRC was partly attributed to the activation of melanoma differentiation-associated protein 5 (MDA5)/MAVS sensing of HERV-derived dsRNA, and subsequent activation of interferon regulatory factor 7 (IRF7) (103). In non-small cell lung cancer (NSCLC) a low-dose DNMT and histone deacetylase (HDAC) inhibitor combination regimen again induced viral mimicry *via* HERV dsRNA upregulation. This led to c-myc downregulation and increased chemokine (C-C motif) ligand 5 (CCL5). *In vivo*, effector T-cell function was restored, preventing tumour-immune evasion (113). The concomitant upregulation of HML-2 (and other) TAAs may, at least in part, underly the anti-tumour effect of epigenetic modifying treatment approaches, therefore, providing a rationale for the complimentary use of anti-HML-2 antibodies or cell therapies. Another means of exploiting HML-2 in cancer therapy could be as an anti-cancer vaccine. Vaccination against HML-2 could prove effective in tumour types where HML-2 reactivation is an early event during carcinogenesis. Kraus et al., constructed a modified vaccinia virus Ankara (MVA) expressing HML-2 Env. Prophylactic vaccination of mice prevented tumour establishment by murine renal carcinoma cells genetically modified to express HML-2 Env (114). However, caution should be observed until a more comprehensive profile of HML-2 expression in all healthy human tissues is reported. HML-2 is known to be expressed in stem cells and the placenta, alluding to a potential role in normal human physiology. Therefore, anti-HML-2 vaccination has the potential to induce autoimmunity. Furthermore, xenograft mouse models that lack inherent HML-2 expression do not allow effective evaluation of potential side effects.

## Conclusion and Future Perspectives

**Figure 2:**
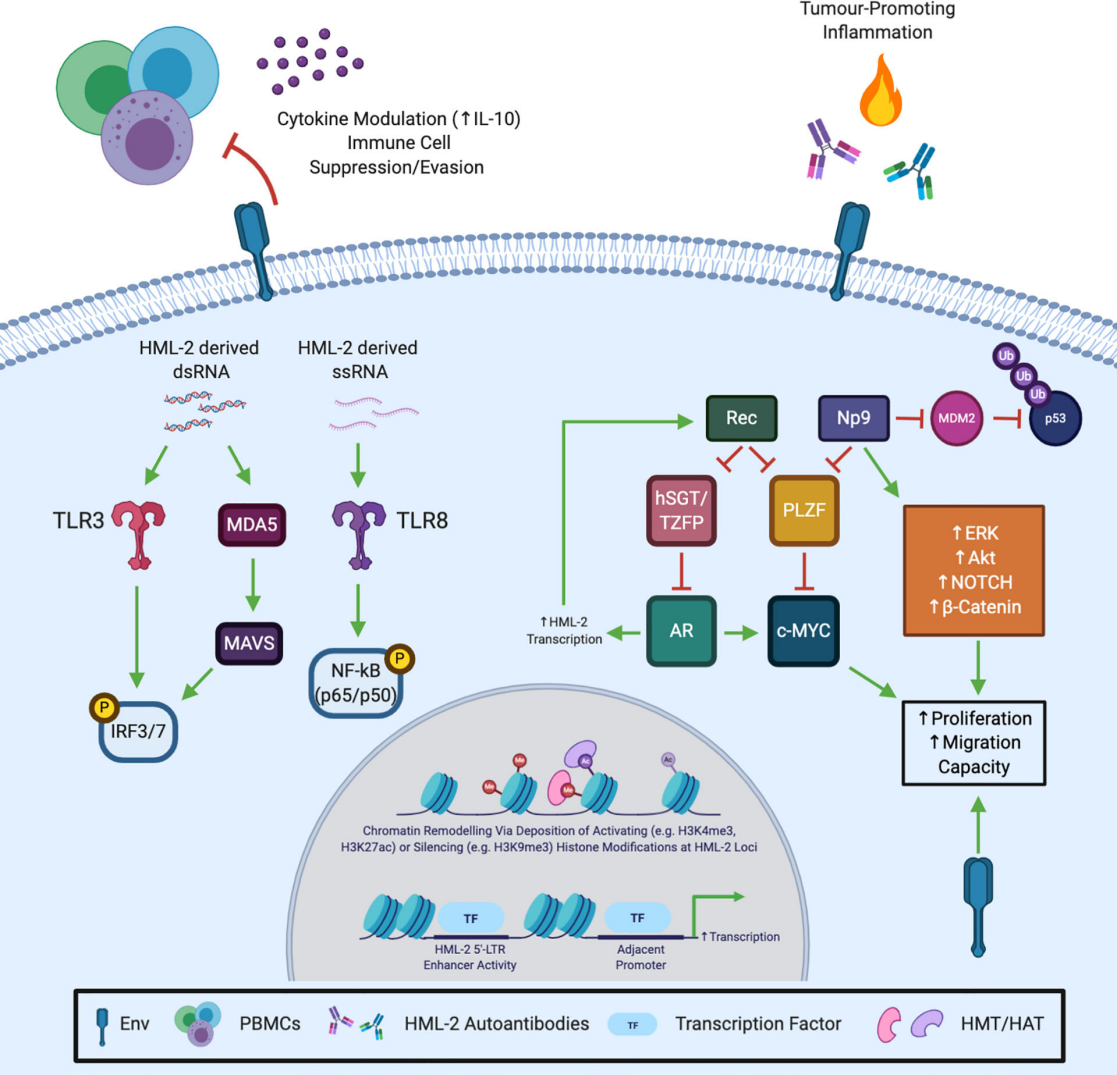
Potential Mechanisms and Molecular Underpinnings of HML-2 Induced Oncogenesis/Tumour Promotion. HML-2 Gag and Env are known to be immunogenic and may contribute to chronic tumour-promoting inflammation. In addition, HML-2 ssRNA binds and activates Toll-like receptor 8 (TLR8), while HML-2 and other HERV-derived dsRNAs may activate Toll-like receptor 3 (TLR3) and the melanoma differentiation-associated protein 5 (MDA5)/mitochondrial antiviral signalling protein (MAVS) pathway to elicit a type I IFN response and nuclear factor -κB (NF-κB) activation. Conversely, Env has been shown to possess an immunosuppressive domain and may inhibit peripheral blood mononuclear cell (PBMC) activation *via* interleukin-10 (IL-10) to aid tumour immune escape. Rec inhibits the androgen receptor (AR) repressor proteins human small glutamine-rich tetratricopeptide repeat (hSGT) and testicular zinc finger protein (TZFP), potentially forming a positive feedforward loop driving chronic HML-2 transcription and AR activation. Np9 has been shown to activate multiple oncogenic signalling pathways (including ERK, Akt, NOTCH and β-catenin), while both accessory proteins have been shown to induce c-Myc transcriptional derepression by inhibiting the promyelocytic leukaemia zinc finger protein (PLZF). Np9 can also bind and inhibit the E3 ubiquitin ligase mouse double minute 2 homolog (MDM2), however, the impact of the resultant p53 stabilisation would likely depend on p53 mutational status. Finally, HML-2 insertions may regulate neighbouring gene expression by (1) recruiting histone methyltransferases (HMT) or histone acetyltransferases (HAT) to regulate chromatin accessibility, or (2) serve as alternative enhancer regions that drive adjacent gene transcription. Created with BioRender.com.

The literature outlined and discussed in this review provide strong evidence that the reactivation of HML-2 during carcinogenesis is not simply an epiphenomenon, but actively contributes to tumour progression. HML-2 expression correlates with disease progression and poor outcome in multiple tumour types. While further validation in larger cohorts is required, HML-2 mRNA/protein expression and autoantibodies may provide robust cancer-specific tools in molecular oncodiagnostics. At the functional level, HML-2 proteins contribute to multiple hallmarks of cancer including tumour growth, metastasis, immune cell evasion and tumour-promoting inflammation (summarised in **Figure 2**). Increasingly more evidence is emerging to suggest at least a partially role for HML-2 in cancer aetiology, however, further research is needed. Furthermore, targeting HML-2 proteins may be a viable new modality for the personalised treatment of aggressive, highly expressing tumour subsets. That said, HML-2 biology is still in its infancy and considerable hurdles exist which are limiting its advancement. Poor annotation in human genome assemblies and their repetitive nature means HML-2 (and other TE) transcripts are excluded from conventional RNA-seq and other bioinformatic analyses (115). High levels of sequence homology (>98%) between evolutionarily young loci provides difficulty in assigning unique reads and must be accounted for to guard against their underrepresentation (116). Nonetheless, multiple groups have developed open source tools for HERV/TE transcript quantification which will allow for a more ubiquitous interrogation of their expression going forward (115–117). HML-2 proteins do not, thus far, appear to have been exapted in normal human physiology, being largely restricted in healthy somatic tissue. A “TEome” or “HERVome” is urgently required to comprehensively catalogue TE/HERV transcript and protein expression in healthy and diseased tissues. This is essential to conclusively unravel and understand their association with disease, and decisively rule out any normal physiological role. At the functional level there is a paucity of information pertaining to the tertiary structure of HML-2 proteins. A greater understanding of the functional motifs present in HML-2 proteins is necessary to aid in the identification of interacting partners and facilitate drug discovery. Finally, owing to the fact HML-2 mRNAs are bioactive and capable of stimulating innate immune receptors, an effort must made to uncouple their effects from their protein products.

## Author Contributions

ED, DB, JM, FK, and SG reviewed the literature, analysed the data, and contributed to writing the manuscript. All authors contributed to the article and approved the submitted version.

## Funding

This publication has emanated from research supported in part by a grant from Science Foundation Ireland (SFI) and the European Regional Development Fund (ERDF) under grant number 13/RC/2073 and grant number 17/CDA/4638, a Breast Cancer Now grant number 2015NovPhD643, a Galway University Foundation Hardiman Research Scholarship, and a Galway University Foundation Rachel Kenneally Triple Negative Breast Cancer Fund, grant number RNR1580.

## Conflict of Interest

The authors declare that the research was conducted in the absence of any commercial or financial relationships that could be construed as a potential conflict of interest.

## Abbreviations

HERV, Human endogenous retrovirus; HML-2, Human MMTV-like 2; TE, Transposable element; LTR, Long terminal repeat; RcRE, Rec responsive element/Regulatory cORF-responsive element.
